# Cellular and Molecular Mechanisms of Recessive Hereditary Methaemoglobinaemia Type II

**DOI:** 10.3390/jcm7100341

**Published:** 2018-10-10

**Authors:** Emilio Siendones, Manuel Ballesteros, Plácido Navas

**Affiliations:** 1 Centro Andaluz de Biología del Desarrollo, Departamento de Anatomía, Fisiología y Biología Celular, Universidad Pablo de Olavide-CSIC, 41013 Sevilla, Spain; mbalsim@upo.es (M.B.); pnavas@upo.es (P.N.); 2 Centro de Investigación Biomédica en Red (CIBERER), Instituto de Salud Carlos III, 41013 Sevilla, Spain

**Keywords:** diaphorase, CYB5R3, methaemoglobinaemia etiology, NAD+ depletion, NAD(P)H oxidase

## Abstract

Cytochrome b5 reductase 3 (CYB5R3) is a membrane-bound NADH-dependent redox enzyme anchored to the mitochondrial outer membrane, endoplasmic reticulum, and plasma membrane. Recessive hereditary methaemoglobinaemia (RHM) type II is caused by CYB5R3 deficiency and is an incurable disease characterized by severe encephalopathy with mental retardation, microcephaly, generalized dystonia, and movement disorders. Currently, the etiology of type II RHM is poorly understood and there is no treatment for encephalopathy associated with this disease. Defective CYB5R3 leads to defects in the elongation and desaturation of fatty acids and cholesterol biosynthesis, which are conventionally linked with neurological disorders of type II RHM. Nevertheless, this abnormal lipid metabolism cannot explain all manifestations observed in patients. Current molecular and cellular studies indicate that CYB5R3 deficiency has pleiotropic tissue effects. Its localization in lipid rafts of neurons indicates its role in interneuronal contacts and its presence in caveolae of the vascular endothelial membrane suggests a role in the modulation of nitric oxide diffusion. Its role in aerobic metabolism and oxidative stress in fibroblasts, neurons, and cardiomyocytes has been reported to be due to its ability to modulate the intracellular ratio of NAD^+^/NADH. Based on the new molecular and cellular functions discovered for CYB5R3 linked to the plasma membrane and mitochondria, the conventional conception that the cause of type II RHM is a lipid metabolism disorder should be revised. We hypothesized that neurological symptoms of the disease could be caused by disorders in the synapse, aerobic metabolism, and/or vascular homeostasis rather than in disturbances of lipid metabolism.

## 1. Introduction

CYB5R3 (NADH: ferricytochrome b5 oxidoreductase; EC 1.6.2.2) deficiency causes a rare recessive hereditary methaemoglobinaemia (RHM). The CYB5R3 gene encodes for two isoforms: a soluble isoform, exclusively expressed in erythrocytes, and a membrane-bound isoform expressed in all cells. Both human isoforms are produced from a single gene locus, *DIA1* (updated to *CYB5R3* (22q13.2) by the Human Genome Organization) [1,2]. Human *CYB5R3* of the non-erythroid cells encodes for an isoform that exhibits an additional exon (M) upstream of the first exon of the soluble protein present in erythrocytes [3]. Therefore, the two enzymes are identical, but the membrane-bound isoform contains a short amino acid sequence (MGAQLSTL), which is a myristoilated anchor at its N-terminus [4,5]. Deficiency of the enzyme can occur as a result of mutations in the CYB5R3 gene and has two clinical phenotypes based on defects in either the soluble or the membrane-bound isoforms. Soluble CYB5R3 is specifically expressed in erythrocytes for methaemoglobin (MetHb) reduction, and its deficiency is responsible for type I RHM, which is a benign condition with mild cyanosis, fatigue, and shortness of breath upon exertion, and high erythrocyte content of MetHb [6,7,8,9]. The membrane-bound CYB5R3 isoform is anchored to the outer mitochondrial membrane (OMM), endoplasmic reticulum (ER), and plasma membrane (PM) of all cells, and its deficiency, causes type II RHM, an incurable encephalopathy with permanent mild cyanosis. 

Nearly 50 mutations in both FAD- and NADH-binding domains have been identified so far, causing truncated CYB5R3 or incorrect mRNA splicing, although most mutations are missense. Truncating mutations leading to the loss of enzymatic activity have been associated with type II RHM, while the missense mutations have been associated with type I RHM, probably because these mutations only induce the instability and the decrease of its concentration in erythrocytes due to the lack of a nucleus [10]. However, there is no a pattern for distinguishing between both types of RHM [8,9,10,11,12,13]. For example, the mutation replacing cysteine-204 by tyrosine (p.Cys204Tyr) produced type I RHM, whereas the replacement of the same cysteine by arginine (p.Cys204Arg) resulted in type II RHM [8]. Further, the same mutation p.Gly76Ser was associated with a type I RHM patient [11] and a type II RHM patient [12], thereby suggesting that genetic, metabolic, or environmental factors could determine the phenotype of each type of RHM. In fact, an atypical intermediate phenotype between types I and II RHM was also reported for several patients with milder neurological symptoms [9,13]. Two of these patients showed a novel mutation replacing arginine by proline (p.Arg58Pro) in the presence of either p.Gly76Ser or p.Leu188del mutations associated with a more severe phenotype that type I but milder than type II RHM [13]. In any case, deficiency of CYB5R3 in non-erythroid cells can lead to a complex spectrum of neurological symptoms currently diagnosed as type II RHM. 

Patients with either type I or II RHM exhibit cyanosis at birth, whereas the clinical neurological phenotype manifests later, after 4–6 months of life. Therefore, early diagnosis is only based on cyanosis symptoms, such as greyish-blue skin or chocolate-brown blood [8]. Encephalopathy with progressive microcephaly and severe brain developmental deficiency is present in all type II RHM patients, which can develop a complex neurological symptomatology: white matter changes on the brain, developmental delay, oral aversion, seizures, dystonia, and dystonia-derived scoliosis, strabismus, failure to thrive, and refractory epilepsy [9,14]. The life expectancy of these patients does not reach beyond 10 years of age [8], although a patient with type II RHM has been reported to have reached 23 years of age [9]. Cyanosis symptoms of RHM can be treated daily with ascorbic acid [15], riboflavin [9], or methylene blue [16]. Ascorbic acid treatment of a type II RHM patient showed positive improvement of some neurological features, such as motor skills [17]; however, there is no effective treatment for the neurological deterioration of type II RHM [9].

## 2. Hypothesis of Demyelination of Neurons in Type II RHM by Disturbances in Lipid Metabolism

The demyelination of neurons as a cause of type II RHM is a broadly accepted hypothesis because CYB5R3 participates in desaturation and elongation of fatty acids, and also in cholesterol biosynthesis. Thus, deficiency of CYB5R3 would lead to abnormal lipid metabolism and probably to concomitant neuronal damage by demyelination. The evidence that CYB5R3 plays a role in lipid metabolism is derived from several studies that focused on the cytochrome b5A (CYB5A) microsomal isoform. CYB5R3 catalyses electron transfer from NADH to diverse substrates, with CYB5A being the most conspicuous. In turn, CYB5A can transfer electrons to multiple acceptors of different metabolic pathways [4], such as cholesterol biosynthesis, fatty acid elongation and desaturation, and P450-mediated metabolism. Chemical inhibition of CYB5R3 induced the decrease of reduced CYB5A levels, cholesterol intermediates [18,19], and desaturated lipids [20]. Additionally, CYB5R3 is a component of the microsomal desaturase complex [21]. Considering this hypothesis, several studies have been published describing the results of an autopsy carried out on a 33-month-old girl who died from type II RHM [22], which showed a reduced content of both cholesterol and phospholipids (80% of normal) in white but not grey matter, and the amount of cerebrosides was 48% of normal in both white and grey matter. The molar proportion of cholesterol to phospholipids was hardly decreased in the myelin, and the cerebroside-to-phospholipids ratio was markedly reduced [22]. Furthermore, the unsaturated/saturated fatty acids ratio was slightly lower compared with healthy controls in both adipose tissue [23] and the liver [24]. These results evidenced an impaired lipid metabolism with an apparent central demyelination linked to type II RHM, but the reduction of unsaturated fatty acids (linoleic, linolenic, and arachidonic acids) was only 10% and the data came from a single patient. However, these analyses strengthened the notion in the following decades that type II RHM is caused by alterations in lipid metabolism. 

## 3. Additional Roles for Membrane-Bound CYB5R3

CYB5R3 has also been shown to be involved in CYP-450-mediated hydroxylation of steroid hormones and xenobiotic drugs [25], contributing to the maintenance of genomic stability by promoting detoxification of xenobiotics through the P450 system in mice [26] and preventing ediethylnitrosamine-induced liver carcinogenesis [27].

It has been broadly shown that an alternative human isoform to microsomal CYB5 is located in the outer mitochondrial membrane (OMM), which can interact with CYB5R3 to form a system responsible for regeneration of mitochondrial ascorbate and the protection of membrane from lipid peroxidation [28,29,30]. Interestingly, CYB5R3 was related to ascorbate regeneration in a patient with type II RHM [10], suggesting that the decreased intracellular ascorbate levels might contribute to the phenotype of the disease. In the 1990s, CYB5R3 was located at the PM for transferring electrons from NADH to coenzyme Q (CoQ), which stabilized extracellular ascorbate [31,32,33], to recycle PM vitamin E [34] and to protect against ceramide-induced apoptosis [35] without CYB5 participation. These components plus the transient contribution of NAD(P)H-CoQ-reductase1 (NQO1) constitute the trans-PM redox system (PMRS), a major antioxidant system against extracellular insults [36]. PMRS has been recently depicted as a neuronal defence centre against oxidative stress by maintaining a stable redox environment required for neuronal viability [37] and preserving the cytosolic NAD^+^/NADH ratio [38]. CYB5R3 is nowadays recognized as a pivotal enzyme in the survival of human neuroblastoma cells undergoing metabolic and oxidative stress [39]. CYB5R3 has a key role in the maintenance of efficient aerobic metabolism [3] and its activity in the PM of rat brains and neuronal cells confers protection against oxidative stress under caloric restriction [40]. ChIP analysis of the human *CYB5R3* gene promoter has recently demonstrated a coordinated action between NRF2 and FOXO3a, two main human regulators of cellular redox homeostasis, in response to environmental stressors and growth factor starvation. This might point towards the interplay between nutrient intake and oxidative stress pathways on cellular homeostasis [3]. Furthermore, it has been proved that CYB5R3 plays a clear role in mitochondrial homeostasis in fibroblasts from type II RHM patients, CYB5R3-silenced human cells, and CYB5R3-overexpressed human neuroblastoma cells [3,39]. These authors suggest that resistance to oxidative stress, improvement of mitochondrial electron transport chain activities, oxygen consumption rate, and ATP production were probably due to the ability of CYB5R3 to modulate the NAD^+^/NADH ratio and maintain cytosolic NAD^+^ levels. Recently, it has been shown that cardiomyocyte-specific inducible *Cyb5r3*-knockout mice that managed to live at least 15 days exhibited decreased mitochondrial size and downregulated mitochondrial biogenesis [41].

CYB5R3 has been also associated with lipid rafts at the PM of cerebellar granule neurons, indicating its role in interneuronal contact sites [42,43]. These authors showed the increase of CYB5R3 protein during in vitro maturation of rat cerebellar granule neurons and they suggested that disturbances of CYB5R3 content in lipid rafts could have a relevant impact on synaptic functionality and neuronal defense. CYB5R3 was abundantly located in the caveolin-protein complex of the vascular endothelial membrane [44], modulating the diffusion and bioavailability of nitric oxide through the α-globin redox state in these cells [45,46]. It has also recently been shown that CYB5R3 activity in vascular smooth muscle cells is involved in the CYB5B-independent reduction of oxidized soluble guanylate cyclase (sGC), which is essential for nitric oxide sensitization, cGMP production, and protein kinase G-dependent signalling activation and vasodilation [47].

## 4. New Insights on the Aetiology of Neurological Disorders Caused by CYB5R3 Deficiency

The involvement of CYB5R3 in the desaturation and elongation of fatty acids, cholesterol synthesis, or CYP-450-mediated detoxification is dependent on CYB5A in the ER [48,49], but there is no clear evidence to demonstrate a similar role in the PM. Noteworthy, redox functions of CYB5R3 in haemoglobin rely on electron transfer through CYB5A [7,50]. Nevertheless, the antioxidant protection of PMRS against lipid peroxidation by CoQ_10_ reduction in calorically restricted brain cells [39,40] or blood vessel dilatation by sGC reduction [46,47] is independent of either CYB5A or CYB5B. In addition, human deficiency of *CYB5A* causes type IV methaemoglobinaemia and ambiguous genitalia [51] but, surprisingly, it does not cause neurological symptoms. Interestingly, unlike *Cyb5r3*, *Cyb5a*-knockout mice survive and do not exhibit a type II RHM phenotype [52,53]. Therefore, the decreased CYB5A-dependent desaturation of fatty acids by CYB5R3 deficiency does not seem be involved in the pathophysiology of type II RHM. On the other hand, the microsomal CYB5R3/CYB5A complex modulates the synthesis of mono- and polyunsaturated fatty acids (PUFAs) through fatty acid desaturases (FADSs) [21,54], which are encoded by *FADS1*, *FADS2*, and *FADS3* genes in the human chromosome 11. The reduction of the desaturation of fatty acids in human deficiency of *FADS2* in a 9-year-old patient induced cheilosis, hyperkeratotic rash over the arms and legs, dystrophic nails, perineal dermatitis, corneal ulcerations and marked photophobia, and brittle hair with a maximum length of only a few centimetres, but the neurological examination was normal [55]. Therefore, disturbances in the elongation and desaturation of fatty acids are mainly involved with skin abnormalities but do not seem to be relevant to the aetiology of type II RHM. Thus, abnormal lipid metabolism, classically reported for CYB5R3 deficiency, is likely to be a secondary phenotype rather than a direct cause of the disease. Indeed, only lipid metabolism impairment cannot explain by itself some manifestations observed in patients, such as the delayed synostosis of the cranial bones or the hypertrophy of the gums and absent or underdeveloped teeth [10]. 

Neuronal injuries and developmental disorders, delay, and disabilities in newborn and children with congenital heart disease are very common [56,57]. It is unclear if the encephalopathy associated with type II RHM is a secondary consequence of heart failure, but interestingly, it was recently reported that cardiomyocytes require CYB5R3 activity to sustain their mitochondrial homeostasis, and their deficiency in the heart leads to death in mice at 15 days after deficiency induction [41]. Therefore, we cannot rule out that a congenital heart disease may be an aetiological mechanism underpinning the encephalopathy of type II RHM. 

Increased NAD^+^ levels by mitochondrial uncoupling, caloric restriction [58], or the prevention of NAD^+^ depletion by nicotinamide [59] have been associated with neuroprotection against excitotoxicity in cerebral ischemia. CYB5R3 helps to prevent NAD^+^ depletion by boosting the cytosolic NAD^+^/NADH ratio [3,39]. Thus, the control of aerobic metabolism and the cytosolic NAD^+^/NADH ratio by CYB5R3 could provide a key regulatory function in the maintenance of neuronal health and, accordingly, a defect of this mechanism of action could be directly related to the neurological disorders in type II RHM. Multiple studies have shown that NAD^+^-consuming enzymes, such as sirtuins, are promising therapeutic targets for neurological diseases [60]. It is unknown if cytosolic NAD^+^ is exhausted in a type II RHM patient brain, but pharmacological or nutraceutical therapy with NAD^+^ precursors, such as nicotinamide mononucleotide [61] and/or a nutritional approach with a ketogenic diet [62] could be implemented to increase intracellular NAD^+^ levels or prevent its possible depletion due to the disease.

Lipid rafts in the synapse are essential microdomains for neuronal signalling of adhesion molecules and guidance receptors [63] and are clearly related to the processes involved in neural development and synaptic plasticity [64]. In the synapse, ascorbate-dependent NADH oxidase activity is increased in the presynaptic vesicles of the PM [65]. Presently, the true magnitude of the role played by CYB5R3 linked to lipid rafts in the synapse is unknown; however, we think the involvement of CYB5R3 in lipid raft signalling mechanisms could be a key point in understanding the aetiology of type II RHM. 

## 5. Conclusions and Future Directions

**Figure 1:**
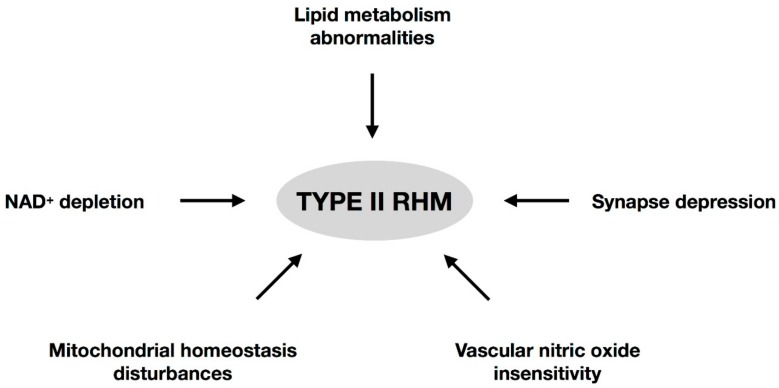
Scheme showing the pathways that could be involved in the etiopathology of the type II RHM.

In spite of the great progress that has been made in studying the roles of CYB5R3 and the unceasing description of multiple and new mutations in its gene, there is insufficient understanding of type II RHM aetiology. In addition, any cure or treatment for the encephalopathy associated with this disease is still non-existent. In this article, we have reviewed a number of relevant studies that show that human deficiency for membrane-bound CYB5R3 shows highly pleiotropic effects. We propose here (Figure 1) that the aetiology of type II RHM cannot only be explained by deficiencies in lipid metabolism, as has been the consensus during the past decades. The knowledge of recently discovered functions of CYB5R3 in mammals, such as its role in cytosolic NAD^+^/NADH regulation, the mitochondrial homeostasis in neurons and cardiomyocytes, the plasticity in synaptic processes, and the vascular vasodilatation response by sGC and protein kinase G-dependent signalling, should be the focus of future studies to better explain all symptoms and to find a possible treatment for this presently incurable disease.
